# Machine Learning-Integrated Explainable Artificial Intelligence Approach for Predicting Steroid Resistance in Pediatric Nephrotic Syndrome: A Metabolomic Biomarker Discovery Study

**DOI:** 10.3390/ph18111659

**Published:** 2025-11-01

**Authors:** Fatma Hilal Yagin, Feyza Inceoglu, Cemil Colak, Amal K. Alkhalifa, Sarah A. Alzakari, Mohammadreza Aghaei

**Affiliations:** 1 Department of Biostatistics, Faculty of Medicine, Malatya Turgut Ozal University, 44280 Malatya, Türkiye; 2 Department of Computer Science, Lakehead University, Thunder Bay, ON P7B 5E1, Canada; 3 Department of Biostatistics and Medical Informatics, Faculty of Medicine, Inonu University, 44280 Malatya, Türkiye; 4 Department of Computer Sciences, College of Computer and Information Sciences, Princess Nourah bint Abdulrahman University, P.O. Box 84428, Riyadh 11671, Saudi Arabia; 5 Department of Ocean Operations and Civil Engineering, Norwegian University of Science and Technology (NTNU), 6002 Alesund, Norway

**Keywords:** explainable artificial intelligence, pediatric nephrotic syndrome, metabolomic biomarker discovery, steroid resistance prediction

## Abstract

**Aim:** Nephrotic syndrome (NS) represents a complex glomerular disorder with significant clinical heterogeneity across pediatric and adult populations. Although glucocorticosteroids have constituted the mainstay of therapeutic intervention for more than six decades, primary treatment resistance manifests in approximately 20% of pediatric patients and 50% of adult cohorts. Steroid-resistant nephrotic syndrome (SRNS) is associated with substantially greater morbidity compared to steroid-sensitive nephrotic syndrome (SSNS), characterized by both iatrogenic glucocorticoid toxicity and progressive nephron loss with attendant decline in renal function. Based on this, the current study aims to develop a robust machine learning (ML) model integrated with explainable artificial intelligence (XAI) to distinguish SRNS and identify important biomarker candidate metabolites. **Methods:** In the study, biomarker candidate compounds obtained from proton nuclear magnetic resonance (1 H NMR) metabolomics analyses on plasma samples taken from 41 patients with NS (27 SSNS and 14 SRNS) were used. We developed ML models to predict steroid resistance in pediatric NS using metabolomic data. After preprocessing with MICE-LightGBM imputation for missing values (<30%) and standardization, the dataset was randomly split into training (80%) and testing (20%) sets, repeated 100 times for robust evaluation. Four supervised algorithms (XGBoost, LightGBM, AdaBoost, and Random Forest) were trained and evaluated using AUC, sensitivity, specificity, F1-score, accuracy, and Brier score. XAI methods including SHAP (for global feature importance and model interpretability) and LIME (for individual patient-level explanations) were applied to identify key metabolomic biomarkers and ensure clinical transparency of predictions. **Results**: Among four ML algorithms evaluated, Random Forest demonstrated superior performance with the highest accuracy (0.87 ± 0.12), sensitivity (0.90 ± 0.18), AUC (0.92 ± 0.09), and lowest Brier score (0.20 ± 0.03), followed by LightGBM, AdaBoost, and XGBoost. The superiority of the Random Forest model was confirmed by paired *t*-tests, which revealed significantly higher AUC and lower Brier scores compared to all other algorithms (*p* < 0.05). SHAP analysis identified key metabolomic biomarkers consistently across all models, including glucose, creatine, 1-methylhistidine, homocysteine, and acetone. Low glucose and creatine levels were positively associated with steroid resistance risk, while higher propylene glycol and carnitine concentrations increased SRNS probability. LIME analysis provided patient-specific interpretability, confirming these metabolomic patterns at individual level. The XAI approach successfully identified clinically relevant metabolomic signatures for predicting steroid resistance with high accuracy and interpretability. **Conclusions:** The present study successfully identified candidate metabolomic biomarkers capable of predicting SRNS prior to treatment initiation and elucidating critical molecular mechanisms underlying steroid resistance regulation.

## 1. Introduction

Nephrotic syndrome (NS) represents one of the most common glomerular disorders encountered in pediatric nephrology practice, characterized by massive proteinuria, hypoalbuminemia, edema, and hyperlipidemia. Nephrotic syndrome demonstrates an annual incidence of approximately 2–7 cases per 100,000 children, with peak occurrence between ages 2–6 years [1,2,3]. Although NS can manifest across all age groups, pediatric presentations exhibit distinctive clinical phenotypes and treatment response patterns that differ substantially from adult-onset disease, necessitating age-specific therapeutic approaches and prognostic considerations [2,4]. The pathophysiology of NS involves disruption of the glomerular filtration barrier, primarily affecting podocytes and the slit diaphragm complex, though the precise molecular mechanisms vary across etiologies. Idiopathic NS, which accounts for approximately 90% of pediatric cases, is broadly categorized based on histopathological findings into minimal change disease (MCD), focal segmental glomerulosclerosis (FSGS), and mesangial proliferative glomerulonephritis, with MCD representing the predominant histological subtype in children [1,2,3,4].

The cornerstone of NS management for over six decades has been glucocorticosteroid (GC) therapy, which induces remission in the majority of pediatric cases. However, a significant clinical challenge arises from the heterogeneous response to this first-line treatment, with approximately 10–20% of children demonstrating primary steroid resistance. Steroid-resistant nephrotic syndrome (SRNS) is defined as the failure to achieve complete remission after 4–6 weeks of daily prednisone or prednisolone at a dose of 60 mg/m^2^/day (or 2 mg/kg/day) followed by 2–5 weeks of alternate-day therapy. This resistance to conventional therapy carries substantial clinical implications, as SRNS is associated with a significantly worse prognosis compared to steroid-sensitive nephrotic syndrome (SSNS), with up to 50% of SRNS patients progressing to end-stage renal disease (ESRD) within 10 years of diagnosis. The molecular mechanisms underlying steroid resistance remain incompletely understood but appear to involve complex interactions between genetic predisposition, immune dysregulation, and podocyte injury pathways [5,6,7].

The clinical consequences of delayed recognition of SRNS are profound and multifactorial. Prolonged exposure to high-dose glucocorticoids in patients who will ultimately prove resistant leads to significant iatrogenic morbidity, including growth retardation, osteoporosis, hypertension, diabetes mellitus, cataracts, and increased susceptibility to infections. Furthermore, the continued progression of glomerular injury in untreated SRNS accelerates nephron loss, potentially compromising long-term renal outcomes. Current clinical practice typically requires 4–10 weeks of glucocorticoid therapy before definitively classifying a patient as steroid-resistant, creating a critical diagnostic window during which inappropriate treatment may cause unnecessary harm while delaying potentially effective alternative therapies. This therapeutic limbo represents a significant unmet clinical need, as early identification of SRNS could enable timely initiation of alternative immunosuppressive regimens such as calcineurin inhibitors, which have demonstrated efficacy in certain SRNS subtypes [8,9,10].

Traditional approaches to predicting steroid resistance have relied primarily on clinical parameters and histopathological examination. Renal biopsy remains the gold standard for definitive diagnosis but is an invasive procedure with inherent risks, particularly in children with significant proteinuria and hypoalbuminemia. Moreover, histopathological findings often lack sufficient predictive value for steroid response, as patients with identical histological patterns may exhibit divergent therapeutic outcomes. Additionally, the high cost and limited availability of comprehensive genetic panels restrict their utility as routine predictive tools in many healthcare settings [11,12].

Metabolomics, the comprehensive analysis of small-molecule metabolites within biological systems, has emerged as a powerful approach for biomarker discovery in complex diseases. This “omics” technology provides a functional readout of cellular processes that reflects the integrated effects of genomic, transcriptomic, and proteomic activity, as well as environmental influences. In renal disease, metabolomic profiling has demonstrated potential for identifying novel pathophysiological mechanisms and diagnostic biomarkers. Previous studies have revealed distinct metabolic signatures in NS patients compared to healthy controls, with alterations in energy metabolism, amino acid pathways, and lipid metabolism. More recently, targeted metabolomic investigations have begun to differentiate between SSNS and SRNS, suggesting that pre-treatment metabolic profiles may contain predictive information about therapeutic response. The application of proton nuclear magnetic resonance (^1^HNMR) spectroscopy for metabolomic analysis offers particular advantages for clinical translation, including minimal sample preparation requirements, high reproducibility, and the ability to quantify a broad spectrum of metabolites simultaneously [13,14,15,16].

The complexity and high dimensionality of metabolomic data present significant analytical challenges that traditional statistical methods often cannot adequately address. Machine learning (ML) techniques have demonstrated remarkable success in extracting meaningful patterns from complex biomedical datasets [17,18], making them particularly well-suited for metabolomic analysis [19,20,21,22]. Supervised learning algorithms can identify subtle, non-linear relationships between metabolite concentrations and clinical outcomes that might be missed by conventional regression approaches. Several studies have applied ML to metabolomic data in renal diseases with promising results. However, the “black box” nature of many high-performing ML algorithms has limited their clinical adoption, as physicians require transparent, interpretable predictions to inform treatment decisions [23,24].

Explainable artificial intelligence (XAI) represents a critical advancement in bridging the gap between high-performance ML models and clinical utility. Unlike traditional ML approaches that provide only outcome predictions, XAI methods such as SHAP (SHapley Additive exPlanations) and LIME (Local Interpretable Model-agnostic Explanations) offer insights into the decision-making process of complex models, identifying which features contribute most significantly to individual predictions. This interpretability is particularly crucial in healthcare applications, where understanding the biological rationale behind predictions can enhance clinical trust, facilitate hypothesis generation, and identify potential biomarkers for further validation. In the context of SRNS prediction, XAI could not only improve diagnostic accuracy but also illuminate metabolic pathways involved in steroid resistance mechanisms, potentially revealing novel therapeutic targets [25,26,27].

Despite the theoretical promise of integrating metabolomics with ML and XAI for predicting SRNS, this approach has not been systematically investigated in pediatric populations. The current study addresses this critical research gap by developing and validating a comprehensive ML-integrated XAI framework for predicting steroid resistance in pediatric NS using pre-treatment plasma metabolomic profiles. The current study selected four ensemble machine learning algorithms for their effectiveness with high-dimensional metabolomic data: (1) Random Forest (RF), an ensemble method robust to overfitting; (2) Extreme Gradient Boosting (XGBoost), a framework known for superior predictive performance; (3) Light Gradient Boosting Machine (LightGBM), an efficient implementation ideal for datasets with numerous features; and (4) Adaptive Boosting (AdaBoost), an iterative method that focuses on misclassified instances. These algorithms were chosen for their complementary strengths in handling challenges like multicollinearity and for their proven success in similar clinical prediction tasks [28,29]. Specifically, we aim to: (1) develop robust ML models capable of accurately distinguishing SRNS from SSNS using baseline metabolomic data; (2) apply XAI techniques to identify key metabolomic biomarkers predictive of SR; and (3) validate these biomarkers through rigorous statistical evaluation and biological plausibility assessment. By achieving these objectives, this research has the potential to transform clinical practice through the development of a non-invasive, pre-therapeutic predictive tool that could guide personalized treatment strategies and improve outcomes for children with nephrotic syndrome. Based on these considerations, we hypothesized that: (H1) a ML model trained on pre-treatment plasma metabolomic data could accurately differentiate SRNS from SSNS prior to therapy initiation; (H2) the integration of XAI techniques would transcend the “black box” limitation of complex models, robustly identifying and ranking key metabolomic biomarkers predictive of SR; and (H3) these biomarkers would demonstrate biological plausibility, elucidating critical molecular pathways involved in treatment failure.

## 2. Results

The comparative performance analysis of four ML models (XGBoost, LightGBM, AdaBoost, and RF) revealed notable differences in predictive accuracy and robustness (in Table 1). RF achieved the highest performance across all metrics, with the best accuracy (0.87 ± 0.12), sensitivity (0.90 ± 0.18), specificity (0.84 ± 0.19), F1-score (0.87 ± 0.19), and AUC (0.92 ± 0.09). It also demonstrated the lowest Brier score (0.20 ± 0.03), indicating superior calibration and reliability in predictions. LightGBM ranked second, showing competitive results, particularly in accuracy (0.85 ± 0.17) and AUC (0.90 ± 0.10), with slightly better specificity than AdaBoost. AdaBoost performed comparably to LightGBM in specificity (0.82 ± 0.22) and AUC (0.89 ± 0.10) but had marginally lower accuracy and F1-score. XGBoost exhibited the lowest performance among the four models, though it still maintained reasonable metrics, such as 0.83 accuracy and 0.89 AUC, suggesting decent predictive capability. Overall, RF emerged as the most robust model, while LightGBM and AdaBoost showed similar performance, all outperforming XGBoost in this evaluation. The consistently low standard deviations in RF’s Brier score and accuracy further highlight its stability compared to other models. The consistently low standard deviations in RF’s metrics (AUC ± 0.09, Brier ± 0.03) further highlight its reliability compared to other models. This stability, coupled with its high sensitivity, makes it particularly suitable for clinical applications where false negatives must be minimized (Table 1).

To statistically validate the superior performance of the RF model, pairwise comparisons with the other algorithms were conducted using paired *t*-tests on the 100 repeated measures. The results, summarized in Table 2, demonstrate that RF’s outperformance was statistically significant. Specifically, RF achieved significantly higher Accuracy, Sensitivity, and AUC compared to XGBoost and AdaBoost (*p* < 0.05), and a significantly higher AUC than LightGBM (*p* < 0.05). Most notably, RF’s superior calibration, reflected by its lower Brier score, was highly significant against all other models (*p* < 0.001). These results formally confirm that RF is the most robust and well-calibrated model for this prediction task.

**Figure 1:**
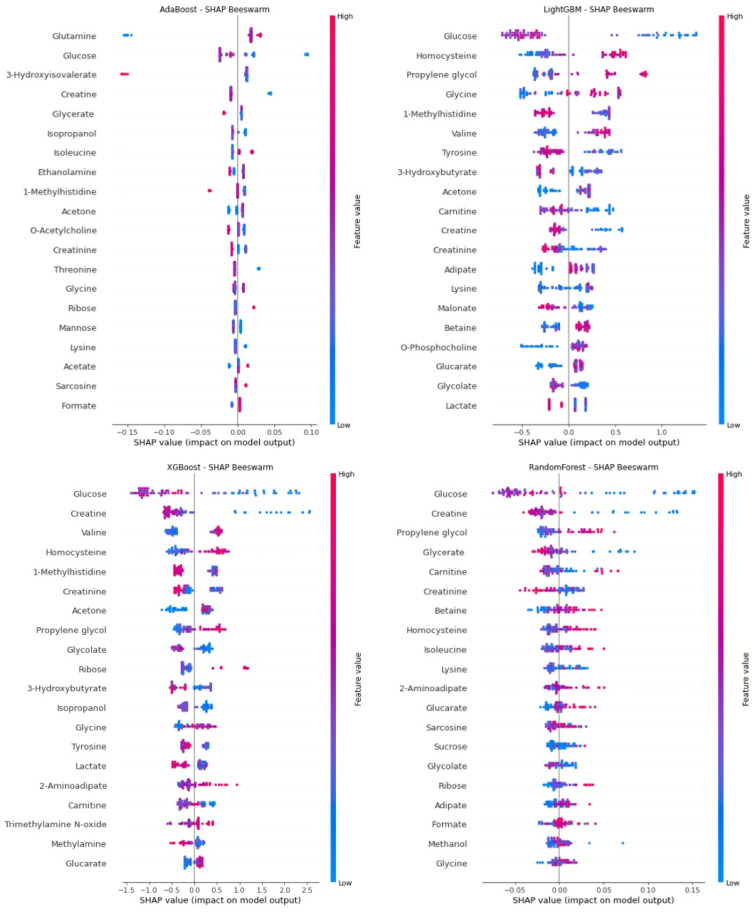
Global Feature Importance for SRNS Prediction: SHAP Summary Plot of Top Lipid Biomarkers.

Figure 1 (SHAP summary plot) shows the global significance of metabolite biomarkers that were inputs to the four ML models used in predicting SRNS. Among the biomarkers appearing in the SHAP beeswarm plots of all models were glucose, creatine, 1-methylhistidine, homocysteine, and acetone, and these biomarkers were consistently identified as significant for SRNS by the ML models. SHAP analysis performed specifically for the RF model with the highest AUC performance further demonstrates the predictive power of these biomarkers. Glucose, the most dominant predictor in terms of both direction and magnitude of SHAP values, was observed to be positively associated with SRNS risk at low glucose levels. Similarly, creatine, glycerate, and creatinine levels exhibited a positive SHAP effect at low concentrations (thereby increasing the probability of SRNS), supporting early biochemical signs of SRNS. Therefore, increasing levels of these compounds to higher levels may have a protective effect or reduce the likelihood of SRNS. Conversely, higher propylene glycol and carnitine levels were observed to be associated with an increased risk of SRNS. These findings, based on SHAP values from the RF model, suggest that, in addition to compounds associated with glucose and creatine metabolism, amino acid derivatives and organic acids may also be considered important biomarkers for predicting SRNS.

**Figure 2:**
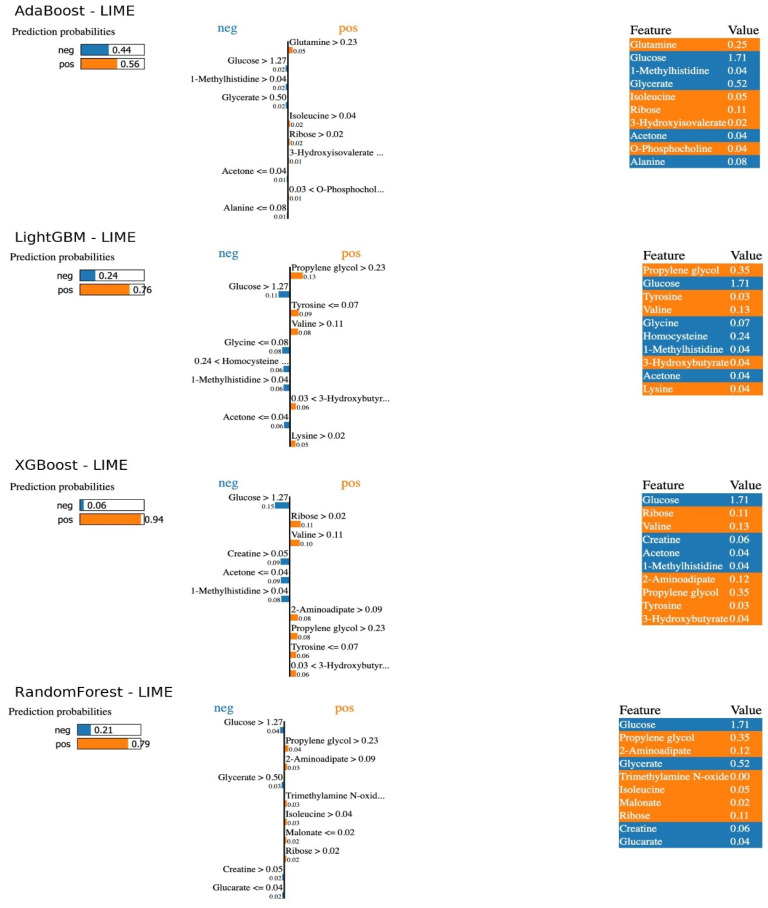
Case-Specific Interpretability of SRNS Predictions Using LIME.

Figure 2 (LIME indicators for individual predictions) provides detailed information about the model decisions for a patient where each model made a positive correct prediction. Each different ML model predicted the patient with different probabilities of having SRNS. The LIME plots explain the conditional statements from which the model made its predictions. Examining the LIME results for the optimal RF model, the patient was classified as having SRNS with a 79% probability and SSNS with a 21% probability. Specifically, the patient’s glucose level being greater than 1.27, glycerol greater than 0.50, and creatinine greater than 0.05 explain the probability ratio resulting in the model classifying SSNS (probability of incorrect class prediction). Furthermore, the patient’s propylene glycol level being greater than 0.23 and 2-aminoadipate greater than 0.09 led the model to assign this patient to SRNS with a higher probability, resulting in a true positive prediction. Consequently, model predictions confirmed the trends obtained from SHAP, demonstrating the importance of low glucose and high propylene glycol levels. The LIME results showed that predictions for high-risk patients were consistently based on markers such as low glucose and high carnitine levels. This individual-level interpretability increases clinical confidence in ML models and supports the potential of these metabolites as early biomarkers (Figure 2).

## 3. Discussion

This study successfully developed and validated an XAI framework integrating ML with metabolomic profiling to predict steroid resistance in pediatric NS prior to treatment initiation. Our results demonstrate that an RF model, trained on pre-treatment plasma metabolomic data, can accurately distinguish SRNS from SSNS with high accuracy (0.87), sensitivity (0.90), and an AUC of 0.92. More importantly, by employing XAI techniques—specifically SHAP for global interpretability and LIME for local, patient-level explanations—we identified and validated a panel of key metabolomic biomarkers, including glucose, creatine, 1-methylhistidine, homocysteine, acetone, propylene glycol, and carnitine, which are critically associated with treatment resistance. This approach addresses a significant unmet clinical need by offering a potential tool for early, non-invasive prediction, which could guide personalized therapeutic decisions and mitigate the risks associated with ineffective steroid exposure and delayed alternative treatment.

In contrast to the current standard of care, which requires 4–10 weeks of steroid treatment to identify resistance, our XAI-integrated model offers a prediction before treatment initiation. Furthermore, while previous metabolomic studies have identified differential profiles between SSNS and SRNS, our study advances the field by not only providing high predictive accuracy but also offering clinically intelligible explanations for why a patient is predicted to be resistant, through biomarkers like glucose and creatine that point to a plausible “energy deficit” hypothesis.

The superior performance of the RF algorithm, compared to other boosting methods like XGBoost, LightGBM, and AdaBoost, can be attributed to its inherent strengths in handling high-dimensional data with potential multicollinearities among metabolite features. RF’s ensemble approach, which builds multiple decorrelated decision trees, is particularly robust against overfitting, a common challenge in studies with a limited sample size relative to the number of features. The model’s high sensitivity is clinically paramount, as the cost of a false negative (misclassifying a resistant patient as sensitive) is exceptionally high, leading to prolonged, toxic, and ineffective steroid therapy. The consistently low standard deviations across performance metrics, especially the Brier score, further underscore the model’s reliability and calibration, suggesting its predictions are not only accurate but also confident [24].

The core of this study’s contribution lies in the application of XAI to elucidate the biological rationale behind the model’s predictions. SHAP analysis transcended the “black box” nature of complex ML models, providing a rank-ordered list of the most influential metabolites. The consistent identification of low blood glucose levels as the strongest predictor of steroid resistance is biologically plausible and aligns with emerging understanding of NS pathophysiology. Podocytes, the key cellular targets in NS, are highly metabolically active and reliant on adequate energy supply, primarily from glucose via aerobic glycolysis. A pre-treatment state of hypometabolism or energy deficit, reflected by low circulating glucose, may indicate podocytes that are already functionally compromised and less able to respond to glucocorticoid-mediated reparative signals. This creates a state of cellular vulnerability where the podocytes are predisposed to injury and less responsive to therapy [7,30].

Similarly, the inverse relationship between creatine levels and SRNS risk offers another compelling metabolic insight. Creatine and its phosphorylated form, phosphocreatine, constitute a critical cellular energy reservoir, particularly in tissues with high and fluctuating energy demands. Low plasma creatine may reflect a depletion of this energy buffering system within renal tissues, specifically podocytes, impairing their ability to maintain the actin cytoskeleton and structural integrity of the glomerular filtration barrier under stress. This energy crisis hypothesis provides a unifying theme, suggesting that SRNS may be characterized by a pre-existing bioenergetic deficit that impedes the cellular response to treatment [15,28].

Beyond energy metabolites, the model identified other significant compounds. The positive association of propylene glycol and carnitine with SRNS risk is intriguing. While propylene glycol is often a solvent in pharmaceutical preparations, its presence as a significant endogenous biomarker warrants further investigation into its metabolic origins. Carnitine is essential for the transport of long-chain fatty acids into mitochondria for β-oxidation. Elevated carnitine levels might indicate a shift in energy substrate utilization from glucose to fatty acids, a compensatory mechanism that may be inefficient or dysregulated in SRNS. Furthermore, the identification of 1-methylhistidine (a product of actin and myosin breakdown) and homocysteine (a marker of oxidative stress and endothelial dysfunction) points toward increased protein catabolism and heightened oxidative stress, both of which are pathways implicated in progressive kidney damage. These findings move beyond prediction and begin to illuminate the complex molecular mechanisms underpinning steroid resistance, suggesting involvement of energy depletion, oxidative stress, and impaired cellular repair [14,29,31].

The LIME analysis powerfully complemented the global SHAP results by providing individualized explanations, which are essential for building clinical trust. For a specific patient, LIME could demonstrate how their low glucose and high propylene glycol levels contributed to their classification as SRNS. This transparency allows clinicians to understand the “why” behind each prediction, moving from a blind trust in an algorithm to an informed assessment of its reasoning based on recognizable metabolic patterns. This patient-centric interpretability is a critical step towards the integration of AI-based decision support systems in real-world clinical workflows, as it facilitates a collaborative dialogue between the physician and the technology [25,27].

In conclusion, this study developed and validated an integrated ML-XAI framework for the pre-treatment prediction of SRNS in pediatric patients. Utilizing plasma metabolomic data, we implemented and compared four ensemble learning algorithms—RF, XGBoost, LightGBM, and AdaBoost—and leveraged SHAP and LIME techniques to identify and interpret key metabolomic biomarkers associated with treatment resistance. Our research successfully addressed its core hypotheses. We demonstrated that: (1) robust ML models can indeed accurately distinguish SRNS from SSNS using baseline metabolomic data, with the RF model achieving superior performance (AUC: 0.92 ± 0.09); (2) pre-treatment metabolomic profiles contain predictive information, and XAI techniques can effectively identify the most influential biomarker candidates, consistently highlighting glucose, creatine, 1-methylhistidine, homocysteine, and acetone across models; and (3) these biomarkers are biologically plausible, pointing towards critical molecular mechanisms underlying steroid resistance, such as a pre-treatment state of cellular energy deficit (low glucose/creatine) and oxidative stress. The primary advantage of this research lies in its novel ML-XAI integration, which moves beyond a “black box” prediction to offer clinically transparent and interpretable results, thereby building a foundation for future clinical trust and utility. The high sensitivity of the model is particularly advantageous for a screening tool, as it minimizes the risk of missing true steroid-resistant cases.

Despite these promising results, some limitations of the study should be acknowledged. The most important limitation is the relatively small sample size (n = 41), which, while sufficient for a robust proof-of-concept study, limits the generalizability of the findings. Furthermore, the fact that the cohort was obtained from a single center increases the risk of potential selection bias. Therefore, validation studies in larger, multicenter, and prospective cohorts are needed to confirm the reliability and external validity of the identified metabolomic signature. In addition, although the proton NMR spectroscopy used in the study provides high reproducibility and simultaneous quantification of a broad range of metabolites, it has lower sensitivity compared to mass spectrometry-based techniques [16]. This may lead to the failure to detect some low-abundance but clinically critical metabolites. Therefore, integrating multi-platform metabolomic data in future studies will enable the creation of a more comprehensive biomarker panel. Another methodological limitation is the use of a 100-repeat repeated holdout approach instead of a nested cross-validation framework. Therefore, a fully integrated cross-validation framework encompassing all preprocessing and feature selection steps is recommended for future research to ensure a more unbiased and reliable assessment of model performance.

## 4. Materials and Methods

### 4.1. Participant, and Analytical Methods

Metabolomic data from pediatric patients with NS, obtained based on plasma samples and publicly available, were used in the current study. The metabolomics dataset used in this study was obtained from metabolomics analyses performed on plasma samples obtained from pediatric NS patients at Nationwide Children’s Hospital (Columbus, OH, USA). The study was conducted in accordance with the principles of the Declaration of Helsinki and was approved by the Malatya Turgut Ozal University Health Sciences Scientific Research Ethics Committee (protocol code = 2025/221, 6 July 2025). Data were analyzed for pediatric NS patients aged 18 months to 18 years with 3+ proteinuria and edema. Plasma samples were collected at disease onset, before any glucocorticoid (GC) treatment. Only patients with definitively verifiable pre-steroid treatment samples were included in the analysis. Clinical response (classification as SRNS (14 patients) or SSNS (27 patients)) was assessed after 6–10 weeks of GC treatment. Plasma samples were analyzed using proton nuclear magnetic resonance (1H-NMR) spectroscopy on a Bruker Avance III 700 MHz spectrometer equipped with a 3 mm cryogenically cooled CRYO QNP probe at 25 °C. A 1D Carr–Purcell–Meiboom–Gill (CPMG) pulse sequence (cpmgpr1d) was used to suppress broad signals from macromolecules. Metabolite identification was achieved by comparison with reference compound spectra and chemical shift databases. Quantification was performed using concentration fitting against a 1 mM internal reference standard. Quality control samples were analyzed periodically to ensure analytical reproducibility. The dataset used in the analyses comprised broad-spectrum 1H-NMR metabolomic levels obtained from pediatric patients with NS [28]. In the current analysis, we used metabolomics panel data specifically obtained from pre-treatment plasma samples to develop predictive models for steroid resistance, ensuring that all metabolomics data reflected baseline metabolic states before glucocorticoid intervention.

### 4.2. Machine Learning Procedure and Explainability

**Figure 3:**
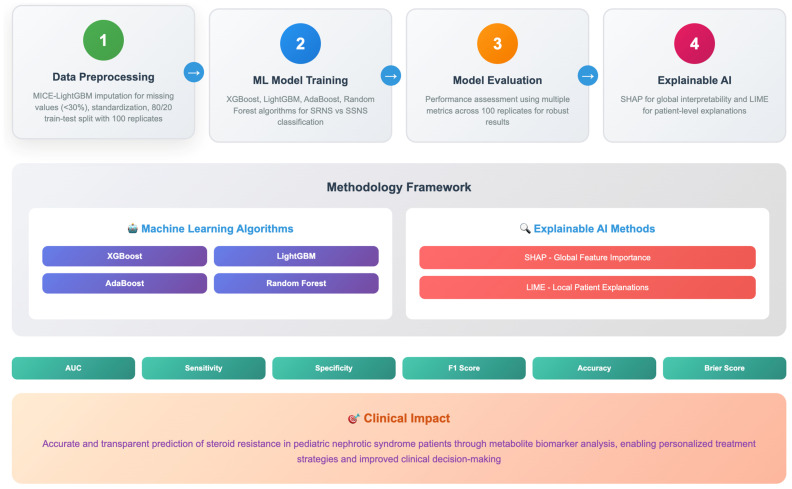
Summary of the methodology workflow.

Missing metabolite values (for features with <30% missingness) were imputed using the Multiple Imputation by Chained Equations (MICE) framework integrated with LightGBM as the imputation engine [7,30]. This approach was implemented using the miceforest Python package (version 5.6.3), which leverages LightGBM’s gradient boosting capability to model complex non-linear relationships between variables during imputation. The imputation procedure utilized the following parameters: five iterations (datasets), mean matching with three candidates, and LightGBM default hyperparameters (100 estimators, learning rate = 0.1, max depth = −1). All available metabolite features were used as predictors in the imputation model to maximize information utilization. This machine learning-based imputation method was selected over traditional approaches (e.g., mean/median imputation) due to its superior performance in preserving complex data structures and relationships in high-dimensional biomedical datasets [7,30]. Metabolite concentration values were standardized to ensure comparability across variables. The dataset was randomly divided into training (80%) and testing (20%), stratified to maintain the original proportion of pediatric patients with SRNS and SSNS. To report unbiased and robust prediction results, we repeated this process 100 times and calculated the mean and standard deviation values for all performance metrics over 100 replicates. Model performance was assessed using AUC, sensitivity, specificity, F1 score, accuracy, and Brier score. Four supervised ML algorithms were applied to develop predictive models: XGBoost, LightGBM, AdaBoost, and RF [32,33,34]. We selected these algorithms because of their strength in handling high-dimensional datasets, potential multicollinearity among metabolites, and non-linear relationships. We fitted performance metrics with 95% confidence intervals derived from 100 bootstrap replicates. To statistically validate the comparative performance of the ML models, pairwise comparisons were made between the best-performing model and three other models. A *p*-value of less than 0.05 was considered statistically significant. A Bonferroni correction was applied to account for multiple comparisons between different performance metrics. SHAP [35,36,37] was applied to the trained ML models to quantify the contribution of each metabolite feature to the prediction of SR in patients with NS. We calculated the mean absolute SHAP values for all patients to rank metabolite biomarkers according to their overall importance in the model for overall interpretability. We also examined the feature impact through SHAP summary and dependency plots, which show both the direction (positive or negative) and magnitude of each metabolite’s effect on SR risk. To ensure biological plausibility, we compared the top-ranked features with previous research to assess their likelihood in the context of SR. For the SHAP analysis, we used the TreeExplainer implementation in the SHAP Python library (version 0.44.1) to calculate feature contributions for all tree-based ensemble models (RF, XGBoost, LightGBM, and AdaBoost). SHAP values were calculated on the test set of each bootstrap replicate to prevent data leakage. Overall feature importance was derived from the mean absolute SHAP values averaged across all samples and 100 bootstrap replicates. The annotator was run with default parameters (feature_dependence = “interventional”, approximate = False), and summary and dependency plots were generated to visualize both the magnitude and direction of metabolite effects on SR risk. LIME [38,39,40] was also used to provide patient-level interpretability by locally approximating complex ML models with simple, interpretable linear models. LIME applied a weighted local surrogate model, recording changes in predicted probability and varying feature values surrounding each selected occurrence. For the LIME analysis, we used the lime_tabular. LimeTabularExplainer class (LIME v0.2.0.1) in regression mode with an exponential kernel width of 0.75. Explanations were generated using the features that contributed most to the local prediction probability. This configuration ensured stable local approximations and reproducible feature weightings. This XAI strategy—SHAP and LIME—increased clinical confidence by ensuring the prediction model was both accurate and transparent, and explained SR prediction results in patients with potential NS. The ML-integrated XAI framework used in the study is presented in Figure 3.

## 5. Conclusions

This study successfully developed an effective ML model, integrated with XAI, for the pre-treatment prediction of SR in pediatric NS. Using metabolomic data, we demonstrated that the Random Forest algorithm provided superior performance, with an accuracy of 0.87 ± 0.12 and an AUC of 0.92 ± 0.09.

The integration of XAI methods like SHAP and LIME was crucial, moving beyond a simple “black box” prediction. These techniques not only confirmed the model’s excellent performance but also offered important new information about the biological basis of the predictions it made. We identified key metabolic biomarkers, such as glucose, creatine, 1-methylhistidine, homocysteine, and acetone, which we were able to identify as being strongly predictive of steroid resistance. The results provide insight into the molecular mechanisms behind therapy response, such as the correlation between elevated risk of SRNS and lower levels of creatine and glucose.

In conclusion, this research offers a non-invasive predictive tool that can accurately identify steroid-resistant patients before treatment even begins. The metabolomic signatures we found are a major step forward for personalized medicine in pediatric nephrotic syndrome. They give us a clear path to develop targeted treatments, which could drastically improve long-term outcomes for these children.

## Figures and Tables

**Table 1:** Comparative Evaluation of XGBoost, LightGBM, AdaBoost, and RF across Classification Metrics.

Metric	XGBoost	LightGBM	AdaBoost	Random Forest
Accuracy	0.83 ± 0.19 (0.792–0.868)	0.85 ± 0.17 (0.816–0.884)	0.84 ± 0.15 (0.810–0.870)	0.87 ± 0.12 (0.846–0.894)
Sensitivity	0.86 ± 0.20 (0.820–0.900)	0.88 ± 0.20 (0.840–0.920)	0.86 ± 0.20 (0.820–0.900)	0.90 ± 0.18 (0.864–0.936)
Specificity	0.80 ± 0.21 (0.758–0.842)	0.82 ± 0.22 (0.776–0.864)	0.82 ± 0.22 (0.776–0.864)	0.84 ± 0.19 (0.802–0.878)
F1	0.83 ± 0.21 (0.788–0.872)	0.85 ± 0.21 (0.808–0.892)	0.84 ± 0.21 (0.798–0.882)	0.87 ± 0.19 (0.832–0.908)
AUC	0.89 ± 0.11 (0.868–0.912)	0.90 ± 0.10 (0.880–0.920)	0.89 ± 0.10 (0.870–0.910)	0.92 ± 0.09 (0.902–0.938)
Brier	0.26 ± 0.12 (0.236–0.284)	0.25 ± 0.07 (0.236–0.264)	0.25 ± 0.03 (0.244–0.256)	0.20 ± 0.03 (0.194–0.206)

Mean ± SD with 95% CI 100 bootstrap replicates in parentheses (lower–upper bounds).

**Table 2:** Pairwise Statistical Comparisons with Random Forest.

Metric	XGBoost vs. RF	LightGBM vs. RF	AdaBoost vs. RF
	(*p*-Value) *	(*p*-Value) *	(*p*-Value) *
Accuracy	<0.05	NS	<0.05
Sensitivity	<0.05	NS	<0.05
Specificity	<0.05	NS	NS
F1	<0.05	NS	NS
AUC	<0.01	<0.05	<0.01
Brier	<0.001	<0.001	<0.001

*: Paired *t*-tests between repeated measures.

## Data Availability

The raw data supporting the conclusions of this article will be made available by the authors on request.
